# A partial mediation effect of father-child attachment and self-esteem between parental marital conflict and subsequent features of internet gaming disorder in children: a 12-month follow-up study

**DOI:** 10.1186/s12889-020-08615-7

**Published:** 2020-04-15

**Authors:** Hyunsuk Jeong, Hyeon Woo Yim, Seung-Yup Lee, Hae Kook Lee, Marc N. Potenza, Sun-Jin Jo, Hye Jung Son

**Affiliations:** 1 Department of Preventive Medicine, College of Medicine, The Catholic University of Korea, 222 Banpodero, Seochogu, Seoul, South Korea; 2 Department of Psychiatry, College of Medicine, The Catholic University of Korea, Seoul, South Korea; 3 Departments of Psychiatry, Neuroscience and Child Study Center, Yale University, New Haven, CT USA; 4 Connecticut Council on Problem Gambling, Wethersfield, CT USA; 5 Connecticut Mental Health Center, New Haven, CT USA

**Keywords:** Internet gaming disorder, Children, Mediation, Self-esteem, Attachment

## Abstract

**Background:**

This study evaluated whether parent-child attachment and self-esteem may mediate the relationship between parental marital conflict and increases in features of internet gaming disorder (IGD) in children at 1 year.

**Methods:**

The baseline and one-year follow-up data for 268 pre-teens aged between 9 and 10 from the Internet User Cohort for Unbiased Recognition of Gaming Disorder in Early Adolescence (iCURE) study were collected. The students were children at low risk for IGD in the initial self-reported assessment, anyone living with both parents, current game user at baseline, and those who completed a 12-month follow-up assessment. The Internet Game Use-Elicited Symptom Screen (IGUESS) was used to identify increases in IGD features at 12 months. To examine a potential mediation effect, structural equation modeling was performed.

**Results:**

The direct effect was statistically significant, and parental marital conflict at baseline significantly predicted the increases in IGD features in children at the 12-month follow-up after adjusting for gender, sex, socioeconomic status, and baseline IGUESS score (ß = 0.206, *P* = 0.003). The indirect effect showed that attachment to fathers through self-esteem was a significant mediating effect (ß = 0.078, *P* = 0.045). Parental marital conflicts were associated with increases in IGD features in children through poor father-child attachment, and in turn, the lower levels of self-esteem in the children.

**Conclusions:**

Parents, especially fathers, should make an effort to bond with their children to reduce the risk of their children’s developing the IGD features.

## Background

Computers, video games, and technological devices are part of most young people’s everyday lives. Prolonged and improper use of the internet and related products may result in harmful effects, including internet gaming disorder (IGD). Children may be particularly vulnerable as their abilities and tendencies to self-regulate are not fully developed, and they are prone to spending prolonged periods playing internet games and using the internet for other purposes [1].

The family typically plays an important role in influencing socialization in the early years of life [2]. The parents’ marital relationship is usually an important influence on child development [3]. Children’s exposure to parental marital conflict may increase risky behavioral and emotional problems [4, 5], including internet addiction in adolescents and young adults [6–9]. While parental marital conflict is a potential risk factor for IGD in children, little is known about the mechanisms underlying the relationship between parental conflict and IGD propensity.

Frequent exposure to high levels of marital conflict may result in poor parent-child attachment [10]. Robust relationships between parents and their children typically promote maturation into emotionally stable, well-adjusted adults, whereas poor attachment relationships may lead to insecurity, anger, and acting-out among children [11, 12]. Thus, parents may contribute importantly to supporting and protecting their child’s psychosocial development by promoting self-esteem through close and warm relationships during childhood [13]. Childhood is a critical period for establishing self-esteem and lower levels of self-esteem have been associated with risky behaviors, such as substance use and delinquency [14–16].

Historically, studies grounded in the attachment theory have focused primarily on the mother-infant relationship, while attachment to the father has been shown to be positively related with higher levels of self-esteem in school-aged children [17]. A previous study reported that roles of father and mother differentially effected on adolescents’ problematic internet use [18].

**Fig. 1:**
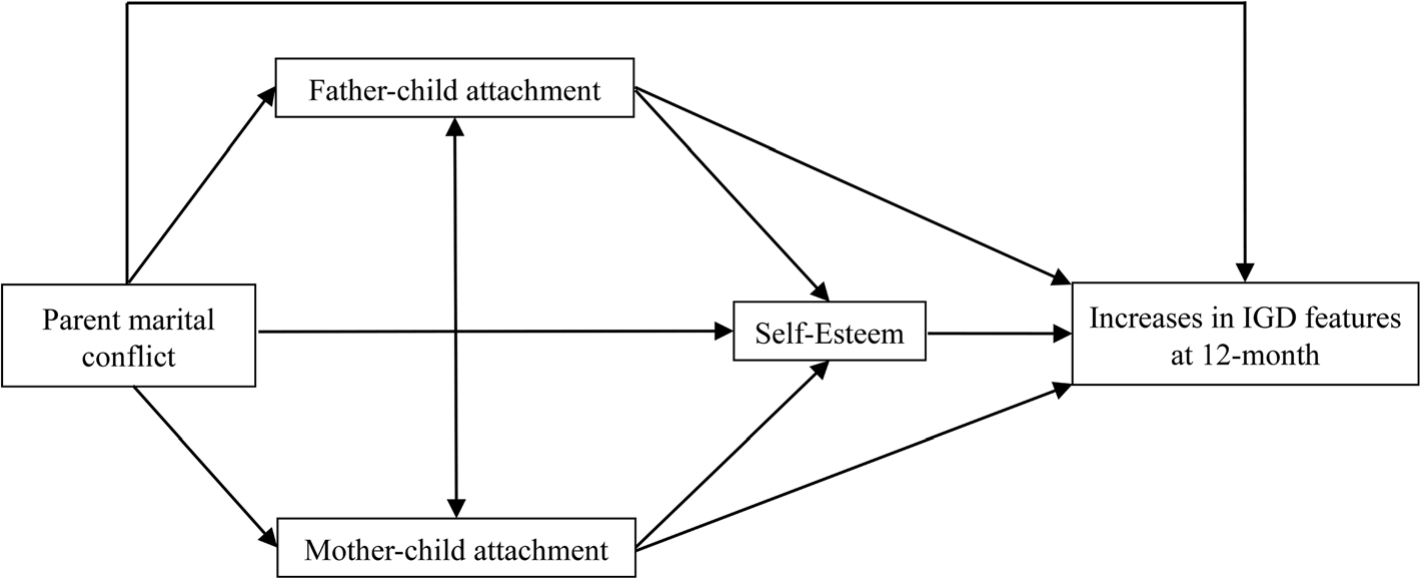
Hypothetical model

The theoretical framework of the study is based on the attachment theory. According to the attachment theory, family harmony and openness were linked with secure attachment [11]. The relationships with parents impact the formation of an internal working model of attachment. Internal working models are generally conceptualized as beliefs on the self and others. Poorer and conflicting family relationship can be affected development of IGD. Game players have been found to depend on gaming to gain self-esteem, to compensate for weak self-image by exhibiting mastery, escaping reality or overcoming difficulties in social interaction [19, 20]. A hypothetical model is presented in Fig. 1.

To the best of our knowledge, no study has investigated the mechanisms connecting parental marital conflict with increases in IGD features over time among pre-adolescent children. We examined whether parent-child attachment via self-esteem at baseline might mediate the relationship between parental marital conflict and increases in IGD features in children from baseline to a 12-month follow-up visit.

## Method

### Participants

This study was conducted using a subsample of the Internet User Cohort for Unbiased Recognition of Gaming Disorder in Early Adolescence (iCURE) study, which is an ongoing prospective cohort study in Korea. Children and adolescents aged 9, 10, and 13 participated in the iCURE study. The protocol for the iCURE study was published previously [21]. Three-hundred-and-ninety-nine pre-teens aged between 9 and 10 participated in the iCURE study from an eligible population of 1061 students at six elementary schools in the cities of Seoul and Uijungbu. To examine possible mediating effects of parent-child attachment through self-esteem in the relationship between parental marital conflict and increases in IGD features in children, we included students who met the following criteria: anyone classified as a “non-case of high risk of IGD” in the initial assessment, anyone living with both parents, and current game use at baseline. A “children at low risk for IGD” was defined as a student with a total score of < 10 in the Internet Game Use-Elicited Symptom Screen (IGUESS) survey. A current game user was defined as having played games over the past year. Of 399 pre-teens aged between 9 and 10, 294 children met the inclusion criteria. To investigate the effect of parental marital conflict on increases in IGD features at 12 months, we excluded 26 participants who did not complete a 12-month follow-up assessment, leaving 268 children in the final analysis.

Written informed consents were acquired from all participants and their parents or legal guardians following explanation of the nature of the principles of research, including confidentiality and the freedom of choice to participate. This study received approval from the Institutional Review Board of The Catholic University of Korea for data analysis (MC19ENSI0001). The iCURE data management board released de-identified iCURE data.

### Measurements

#### Data collection

Data collection was conducted at the above-mentioned schools during school hours. In the baseline assessment, pre-teens aged between 9 and 10 completed the questionnaires in a class setting; a research assistant read the questions with a standard script to help with comprehension and minimize time demands. In the 12-month follow-up assessments, all students completed the questionnaires on their own, using a web-based self-administration method, with a supervising research assistant available to answer questions. Average times spent playing internet games during weekdays and weekends (days and minutes) were obtained from the iCURE study baseline data. One of each child’s parents completed a demographic questionnaire at the participant’s home or at a private space at the school, according to the participant’s preference. The parental questionnaire was administered by trained interviewers at the baseline interview in which, the parents’ year of education, employment status, and socioeconomic status (SES) data were obtained.

#### Parental marital conflict

Level of parental marital conflict was measured by the Children’s Perception of Intraparental Conflict Scale (CPIC), which was developed by Grych, Seid and Fincham (1992) [22]. We used a Korean version of the CPIC which demonstrated good reliability and validity for assessing parental marital conflict at baseline [23]. Although the full version of the CPIC consists of 49 questions, we only adopted the characters of conflict subscale, which contained 19 items [24] and included four dimensions: conflict frequency (four questions), conflict intensity (five questions), (lack of) conflict resolution (seven questions), and stability of conflict (three questions) to measure children’s perception of their parents’ marital conflicts by conducting a confirmatory factor analysis. The response format was a five-point Likert scale ranging from 1 (never) to 5 (always). Total scores ranged from 19 to 95. Cronbach’s alpha is 0.90 for this subscale.

#### Parent-child attachment

Parent-child attachment was measured by the Inventory of Parent and Peer Attachment-Revised version (IPPA-R) [25] at baseline. The questionnaire was developed to assess children’s perceptions of the positive and negative affective/cognitive dimensions of their relationship with their parents, and the questionnaire aimed to be a strong measure of psychological security. We used a Korean version of the IPPA-R in the current study [26]. The IPPA-R is a 25-item questionnaire with responses on a five-point scale, ranging from 1 (“almost never or never true”) to 5 (“almost always or always true”), with higher scores indicating stronger attachment. Children reported on father-child attachment (25 questions) and mother-child attachment (25 questions) separately. The Cronbach’s alphas of the IPPA-R in the original version were 0.87 in mothers and 0.89 in fathers, and in the present study they were 0.93 and 0.93, respectively.

#### Self-esteem

Rosenberg’s self-esteem scale has 10 items, each rated on a five-point Likert scale, ranging from 1 (strongly disagree) to 5 (strongly agree) [27], with a total score range of 10 to 50. Rosenberg defined self-esteem as an individual’s sense of worthiness, which integrates self-respect and self-confidence. This measure detects feelings of self-acceptance, self-respect, and generally positive self-evaluation. Higher scores indicate greater feelings of self-acceptance, self-respect, and generally positive self-evaluation. We used a Korean version of the self-esteem scale which validated good reliability and validity for testing self-esteem at baseline [28]. The Cronbach’s alpha was 0.85 in this study.

#### High risk of internet gaming disorder

Self-reported IGD features were assessed by the IGUESS at both baseline and 12-month follow-up. This instrument was created based on the nine IGD criteria established by Diagnostic and Statistical Manual of Mental Disorders (DSM–5). Students were instructed to respond reflecting their gaming behavior within the last 12 months, with each item rated on a four-point scale: 0 = strongly disagree, 1 = somewhat disagree, 2 = somewhat agree, 3 = strongly agree). A higher score indicates greater IGD severity [29]. A Cronbach’s alpha of 0.80 was observed in this study.

#### Covariates

Possible confounding factors, including gender, age, family SES, and baseline IGUESS score, were obtained from the iCURE baseline data to control for these variables in the final model. The SES data were obtained from parents’ self-evaluations using a seven-point visual analog scale from very low (1) to extremely high (7). SES was reclassified into the lower level of SES (from 1 to 3) and the higher level of SES (from 4 to 7).

### Statistical analysis

Descriptive and correlation analyses were performed using SAS 9.4 (SAS Institute Inc., Cary, NC, USA). Descriptive data were summarized with numbers and percentages for the categorical variables or mean ± SD and ranges for the continuous variables. Structural equation modeling (SEM) was conducted to examine the measurement and mediation models using the Analysis of Moment Structures, version 23.0. (IBM Inc., Chicago, IL, USA). In a confirmatory factor analysis, convergent and discriminant validity examine the extent to which measures of a latent variable share their variance and how they are different from others, respectively. The criterion of Fornell–Larcker (1981) is commonly used to assess the degree of shared variance between the latent variables of the model [30]. According to this criterion, convergent validity is assessed by means of: (1) factor loadings (standardized regression weights), (2) reliability (Cronbach’s alpha), and (3) average variance extracted (AVE). The last entity measures the level of variance captured by a construct versus the variance due to measurement error. Values above 0.7 are considered very good and those between 0.7 and 0.5 is acceptable. Construct reliability (CR) measures whether a set of indicators representing a construct are consistent in their measurement, and it is customary to use the Cronbach’s alpha [31]— values above 0.7 demonstrate that a scale is internally consistent — for this purpose. In addition, we resolved to estimate the CR, which is a less biased estimate of reliability with an acceptable value of 0.7 and above. According to the Fornell–Larcker testing system, discriminant validity can be assessed by comparing the amount of the variance capture by the AVE construct and the shared variance with other constructs (standard error). The levels of the AVE for each construct should be greater than the squared correlation involving the constructs.

A bootstrapping procedure was used to test and verify the paths and indirect effects for statistical significance with bias-corrected methods [32]. A model fit was assessed using the multiple fit indices in terms of absolute fit, incremental fit, and parsimony fit indices. The absolute fit indices included a chi-square ratio over the degrees of freedom (x^2^/df), the goodness-of-fit index (GFI), and the root mean square error of approximation (RMSEA). The incremental fit indices were assessed using the Tucker–Lewis Index (TLI), the normed fit index (NFI), and the comparative fit index (CFI). The adjusted goodness-of-fit index (AGFI) was used for parsimony fit indices. SEM literature suggests that a model fit is good when x^2^/df ≤ 3; CFI ≥ 0.90, TLI ≥ 0.90, GFI ≥ 0.90, NFI ≥ 0.90, RFI ≥ 0.90, AGFI ≥0.90, and RMSEA ≤0.06 [33]. If the path was not statistically significant in the full hypothetical model, we deleted the insignificant path in the alternative model. We compared the model fit index of the two models and adopted the final model with paths that fit significantly better than others.

## Results

### Study population

Of the 268 children, 125 (46.6%) were girls. Among the parents, 180 (67.2%) of the fathers and 177 (60.1%) of the mothers had 13 or more years of education. Most fathers (98.9%) and mothers (59.7%) were employed. For parent-reported family socioeconomic level, 75.7% of participants reported higher levels of SES. The average time spent on internet gaming was 39.4 ± 50.7 min per day during weekdays and 59.9 ± 74.8 min per day during weekend days (Table 1).

**Table 1:** Children and parents information of 268 participants

Variables	n (%) or mean ± SD
Age (years)	9.4 ± 0.6
Gender	
Male	143 (53.4)
Female	125 (46.6)
Years of father’s education level	
0–12	88 (32.8)
≥ 13	180 (67.2)
Years of mother’s education level	
0–12	91 (33.9)
≥ 13	177 (60.1)
Unemployment	
Father	3 (1.1)
Mother	103 (40.3)
Perceived socioeconomic status	375 (16.2)
Low	65 (24.3)
Middle to high	203 (75.7)
Average time spent playing Internet game during weekdays (minutes/day)	39.4 ± 50.7
Average time spent playing Internet game during weekend (minutes/day)	59.9 ± 74.8

### Descriptive statistics in the variables included in the SEM

Correlations among indicators ranged from r = 0.27 to 0.63 and all of their coefficients were significantly correlated (*P* < 0.001). A negative correlation was found between parental marital conflict and parent-child attachment. Both attachments to father and to mother were positively correlated with self-esteem. IGD features at 12 months were positively correlated with parental marital conflict and negatively correlated with parent-child attachment and self-esteem. Children raised in a family with marital conflict may be at risk of developing IGD features (Table 2).

**Table 2:** Means, standard deviations, and correlation coefficients for variables

	1	2	3	4	5	Mean	SD	Range
1. Parent marital conflict	1					27.6	7.9	18–52
2. Attachment to father	−0.49^*^	1				81.5	13.3	46–100
3 Attachment to mother	−0.42^*^	0.59^*^	1			96	13.1	20–100
4. Self-esteem	−0.41^*^	0.58^*^	0.63^*^	1		29.9	4.8	9–36
5. Increases in IGD features^a^	0.27^*^	−0.27^*^	−0.27^*^	−0.38^*^	1	2.7	3.0	0–18

^*^p < 0.001

^a^*IGD* Internet gaming disorder

### Confirmatory factor analysis and convergent and discriminant validities

In consistent with previous conceptualizations of parental marital conflict and attachment instruments, we created a single latent factor model of marital conflict using four indicators, in accordance with the scores on each of the four subscales measures of conflict frequency, intensity, stability, and lack of resolution. Both father-child attachment and mother-child attachment included the two subscales of acceptance and compliment. Self-esteem consisted of positivity and confidence indicators. All measurement models in our sample data were adequate. The Cronbach’s alphas ranged from 0.71 to 0.94 in the measurement subscales. All factor loadings turned out to be greater than 0.5 and convergent validity of the model met the Fornell–Larcker criterion. All the AVE values were above 0.7, indicating a very good fit. Both the Cronbach’s alpha coefficients and CR values were considered acceptable with the values above 0.7 (Table 3).

**Table 3:** Summary of statistics of confirmatory factor analysis

Measurement	Construct	Items	Cronbach’s Alpha	Standardized estimate	AVE^a^	CR^b^
Parent marital conflict	Conflict irresolution	6	0.78	0.61	0.9272	0.9869
	Conflict intensity	5	0.71	0.58	0.9297	0.9845
	Conflict frequency	4	0.71	0.66	0.9523	0.9875
	Conflict stability	3	0.75	0.69	0.9617	0.9868
Attachment to father	Acceptance	18	0.94	0.67	0.9197	0.9932
	Compliment	7	0.71	0.51	0.7938	0.8851
Attachment to mother	Acceptance	18	0.95	0.71	0.9362	0.9951
	Compliment	7	0.67	0.51	0.7188	0.8363
Self-esteem	Positivity	6	0.82	0.51	0.9251	0.9841
	Confidence	4	0.74	0.69	0.9325	0.9821
Increases in IGD features^c^	9 IGD criteria	9	0.81	0.57	0.9518	0.9942

^a^AVE: average variance extracted

^b^CR: construct reliability

^c^IGD: Internet gaming disorder

### Mediating roles of father-child and mother-child attachment and self-esteem on the association between parent marital conflict and risk of IGD

In the analysis of the full proposed hypothetical model, standardized regression coefficients were not statistically significant in the three paths, including “parental conflict → self-esteem,” “attachment to father → increases in IGD features,” and “attachment to mother → increases in IGD features” (Table 4). We tested the alternative model after dropping the three insignificant paths in the hypothetical model. Table 5 summarizes the results of the model comparisons. The alternate model showed a relatively reasonable goodness-of-model-fit in the entire index (x^2^/df = 1.504, GFI =0.953, RMSEA =0.043, TLI =0.951, NFI =0.925, CFI =0.973, AGFI =0.921). The absolute fit indices, incremental fit indices, and parsimony fit indices were satisfied. These results indicated a significant role of attachment to parents and self-esteem in the mechanism explaining the relationship between parental marital conflict and increases in IGD features in children (Table 5).

**Table 4:** Standardized regression estimates of each measurement for the hypothetical model

Path			Standardized regression estimate	*P* value
Attachment to father	←	Parental conflict	−0.618	***
Attachment to mother	←	Parental conflict	−0.462	***
Self-esteem	←	Attachment to father	0.663	***
Self-esteem	←	Attachment to mother	0.258	0.043
Self-esteem	←	Parental conflict	0.064	0.468
Conflict stability	←	Parental conflict	0.797	
Conflict frequency	←	Parental conflict	0.713	***
Conflict intensity	←	Parental conflict	0.643	***
Conflict irresolution	←	Parental conflict	0.849	***
Acceptance	←	Attachment to father	0.806	
Compliment	←	Attachment to father	0.604	***
Acceptance	←	Attachment to mother	0.459	
Compliment	←	Attachment to mother	0.938	***
Positivity	←	Self-esteem	0.898	
Confidence	←	Self-esteem	0.614	***
Increases in IGD features	←	Parental conflict	0.239	0.010
Increases in IGD features	←	Self-esteem	−0.307	0.064
Increases in IGD features	←	Attachment to father	0.084	0.674
Increases in IGD features	←	Attachment to mother	0.061	0.572

^***^P < 0.001

**Table 5:** The goodness-of-fit indices in the two models

	x^2^/df	GFI	RMSEA	TLI	NFI	CFI	AGFI
Hypothetical model	3.818	0.886	0.103	0.781	0.801	0.841	0.818
Alternate model	1.504	0.953	0.043	0.951	0.925	0.973	0.921

*df* degree of freedom, *GFI* goodness-of-fit index, *RMSEA* root mean square error of approximation, *TLI* Tucker–Lewis index, *NFI* normed fit index, *CFI* comparative fit index, *AGFI* adjusted goodness-of-fit index

### Final structural equation model

**Fig. 2:**
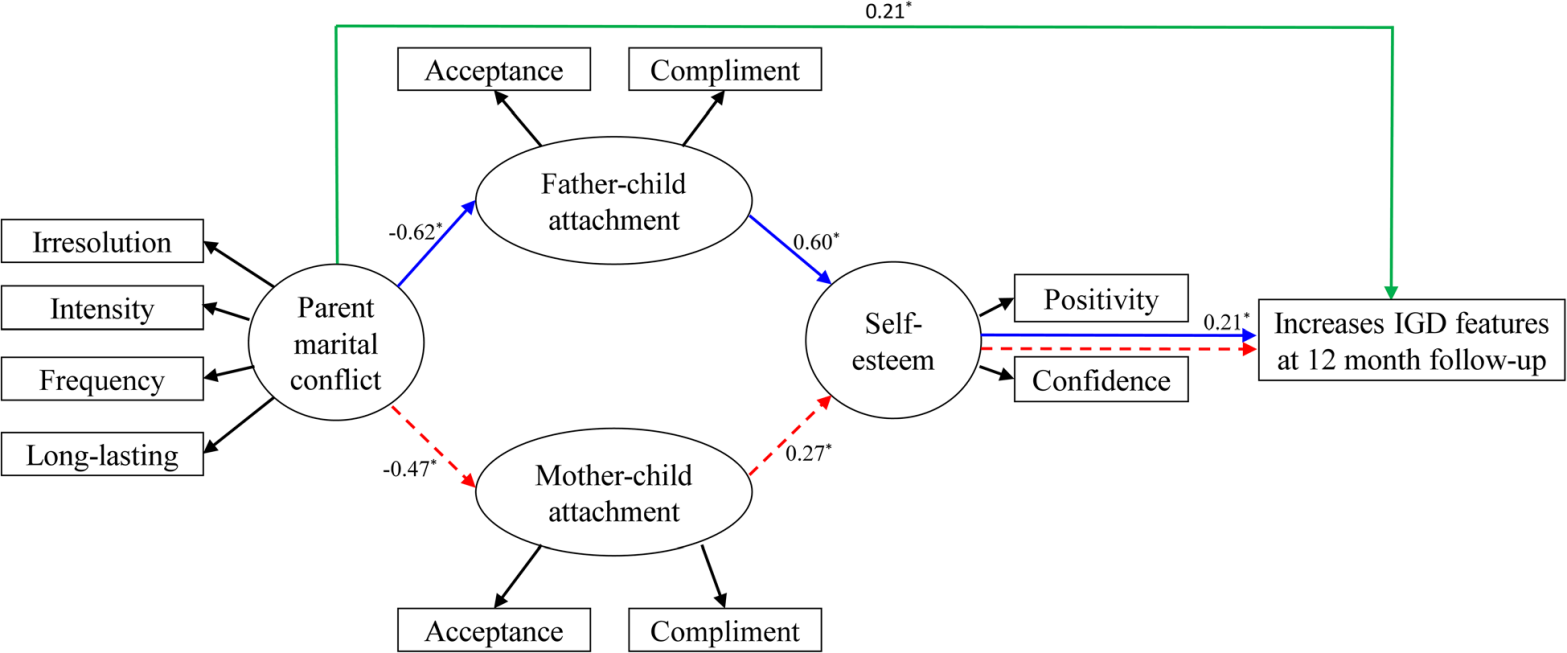
The final model. The partial mediating influence of father-child attachment and off-spring’s self-esteem on the relationships between parent marital conflict and increases in IGD features. All pathways are standardized coefficients. ^*^P < .05. Green lines indicate the direct effect of parental conflict on increases in IGD features (ß =0.206, *P* = 0.003). Blue lines indicate the indirect path for the role of father in self-esteem (ß =0.078; − 0.62 × 0.60 × 0.21, *P* = 0.045). Red dotted lines indicate the indirect path for the role of mother in self-esteem (ß =0.027; − 0.47 × 0.27 × 0.21, *P* = 0.081).

In Fig. 2, standardized path coefficients are displayed in the final SEM, which demonstrates adequate goodness-of-fit measures (x^2^/df = 1.504, GFI =0.953, RMSEA =0.043, TLI =0.951, NFI =0.925, CFI =0.973, AGFI =0.921). This model revealed that parental marital conflict was positively correlated with increases in IGD features and negatively associated with parent-child attachment. Attachment was positively associated with self-esteem, and self-esteem was negatively associated with increases in IGD features. The direct effect of parental conflict on increases in IGD features was statistically significant (ß =0.206, *P* = 0.003). The indirect path for the role of father in self-esteem on the relationship between parental conflict on increases in IGD features was statistically significant (ß =0.078; − 0.62 × 0.60 × 0.21, *P* = 0.045). However, the indirect path for the role of mother in self-esteem was not statistically significant (ß =0.027; − 0.47 × 0.27 × 0.21, *P* = 0.081).

### Direct effect, indirect effect, and total effect

In the alternate model, the direct effect was statistically significant, and parental marital conflict at baseline significantly predicted the increases in IGD features at 12-month follow-up after adjusting for gender, age, SES, and baseline IGUESS score (ß =0.206, *P* = 0.003). The indirect effect showed that attachment to father and self-esteem had significant mediating effects (ß =0.078. *P* = 0.045). However, attachment to mother and self-esteem had not statistically significant mediating effects (ß =0.027, *P* = 0.081) in children. The effect of parental conflict on increases in IGD features was partially mediated by father-child attachment through self-esteem (Table 6).

**Table 6:** Total, direct, and indirect effects in the final structural equation model

Pathways in the final structural equation model	Total effect	Direct effect	Indirect effect
Parent marital conflict ➔ attachment ➔ self-esteem ➔ Increases in IGD features	0.301^***^		
Parent marital conflict ➔ Increases in IGD		0.201^***^	
Parent marital conflict ➔ attachment to father ➔ self-esteem ➔ Increases in IGD			0.076^*^
Parent marital conflict ➔ attachment to mother ➔ self-esteem ➔ Increases in IGD			0.026

^*^P < 0.05, ^**^P < 0.01, ^***^P < 0.001; all *P* values calculated using the bias-corrected bootstrap procedure

## Discussion

The present study examined the roles of parents-child attachments and self-esteem in mediation between parental marital conflict and increases in IGD features among pre-teens aged between 9 and 10. Our findings show that marital conflict at baseline was positively associated with increases in IGD features in children 12 months later, and this association was partially mediated by father-child attachment and self-esteem. This finding suggests that the child’s attachment to a father may play a more important mediating role than attachment to a mother in the association between marital conflict and increases in IGD features in children.

Our findings show a significant direct effect in the association between parental marital conflict and increases in IGD features in children. Perceived marital conflict by children has been associated with addictive online behavior [34, 35]. Thus, children raised in a more conflict-prone family may be in danger of developing gaming problems. It is possible that, because children may not perceive parents’ conflicts as manageable events, they may try to escape the stressful situations by spending more time in the virtual environments of online games. Similar findings have been reported regarding such problematic childhood behaviors as delinquency and substance abuse [36, 37], problematic alcohol use [35, 38], and maladjustment [39].

The current study shows that self-esteem, coupled with father-child attachment, partially mediates the association of parental marital conflict with increases in IGD features in children. Parental marital conflict may cause poor father-child attachment, which in turn, leading to lower levels of self-esteem. Several studies have emphasized the father’s role in the development of healthy emotional functioning in children. Attachment to fathers has been positively related to higher levels of self-esteem [40]. Furthermore, children with positively involved fathers show more psychological adjustment, perform better in school, display less antisocial behavior, and have more successful intimate relationships than children with uninvolved fathers, or those with negatively involved fathers [17, 41]. When children have a quality relationship with their fathers, there is an increased likelihood that their adult life will be happy, satisfying, and characterized by lower degrees of psychological distress [42]. In line with Bowlby’s attachment theory of working models, when a child sees the parent as responsive, accepting, available, and independence-encouraging, a secure attachment develops and the children evaluate themselves as worthy. Furthermore, securely attached adolescents show higher levels of self-esteem than insecurely attached ones [25, 43].

In terms of the indirect effect, parental marital conflict could result in poor attachment to fathers and low self-esteem, with a subsequent impact on increases in IGD features in children. However, mother-child attachment and self-esteem do not appear to mediate the association between parental marital conflict and increases in IGD features. These findings are consistent with the results of previous longitudinal research [13, 44]. The findings could be interpreted in terms of the Bowlby’s attachment theory, in which affective bonds between infant and caregiver, who is typically a mother, form during infancy. Considering that the participants in our research are elementary school students, it is possible that marital conflict negatively affects the attachment to a father and self-esteem. A literature review of previous studies seem to suggested that the relationship with father’s could prevent IGD in adolescents [6]. Good father-child relationship was the most important factor preventing problematic internet use in Chinese adolescents [13]. Father-child relationship (but not mother-child relationship) played an important role in the relationship between parental monitoring and IGD in Chinese adolescents. A previous study examined the different impacts of mother- and father-child relationships on IGD [44]. Unlike infancy, fatherhood influenced adolescent mental health more than motherhood. Father-child communication style plays an important role in the relationship between adolescent aggressiveness and risk of IGD in Korea [45]. Those finding in line with our results in upbringing of father’s role in adolescents’ development.

A previous study reported on the mediating role of parent-child attachment between parental marital conflict and internet addiction in Chinese adolescents [46]. These findings suggested that father-child attachment be more important than mother-child attachment on developing Internet addiction. Thus, those having attachment problems might resort to Internet gaming to avoid the emotional distress caused by attachment situations or to compensate their adverse relationship experiences [47].

There were several limitations of this study. First, of the 1061 eligible students at six elementary schools, only 399 (37.6%) consented to participate in the iCURE study. Therefore, the results may not be reflective of all elementary school students in this jurisdiction. However, the demographic profile of our sample and daily internet gaming time were similar to those of Korean children based on the nation’s annually reported data [48]. Second, because the information was collected through self-administered questionnaires, we cannot rule out an over- or under-reporting in responding to the gaming-related or other questions. According to a previous finding conducted in a subsample of the iCURE study, the false-negative rate and the false-positive rate for self-reported IGD assessment was 44 and 9%, respectively [49].

Third, at the time of the baseline assessment, the participants too young to perform web-based survey by themselves because they were not used to the data collecting method. A research assistant read the questions to help them with comprehension and to minimize time demands at the baseline assessment. In the 12-month follow-up assessments, the participants were able to complete the questionnaires on their own, using a web-based self-administration method, with a supervising research assistant available to answer questions. Compared to the self-administered method, there is a possibility of underestimation in the evaluation of IGD when the research assistant reads the questionnaire and answers it. However, the size of the underestimation would not have been large because the participants were pre-teens aged between 9 and 10.

Fourth, it is possible that the mother-child attachment mediating effect may not be reliable because of the small sample size. However, it seems that the mediating effect of father-child attachment through self-esteem was a more important mechanism than mother-child attachment in the association between parental marital conflict and increases in IGD features. Despite these limitations, there were several strengths of the current study. We used the 12-month follow-up cohort data to examine the directional hypotheses among relationships between parental marital conflict, parent-child attachment, self-esteem and increases in IGD features, using children without IGD and with current internet gaming use at baseline.

## Conclusion

Parental marital conflict was associated with increases in IGD features in children in part through the effects of father-child attachment, and in turn, self-esteem. Higher levels of marital conflict may lay steppingstones to poor attachment with fathers, leading to lower levels of child self-esteem, which may influence children’s problematic gaming behaviors. Our findings show that father-child attachment contributes importantly to self-esteem in children and that both father-child attachment and self-esteem may be important factors in controlling the incidence of IGD features. In the public health perspectives, the results of present study suggest that parents should be careful not to fight with each other at home to protect their children against having IGD. Also, health behavior interventions might benefit from a focus on both individuals’ styles of attachment to father with the hope of increasing more secure attachments and increased availability of self-esteem for children under parents’ marital conflicts.

## Data Availability

The datasets generated during and/or analyzed during the current study are available from the corresponding author.

## Abbreviations

IGD: Internet gaming disorder
iCURE: Internet User Cohort for Unbiased Recognition of Gaming Disorder in Early Adolescence
IGUESS: Internet Game Use-Elicited Symptom Screen
SES: Socioeconomic status
CPIC: Children’s Perception of Intraparental Conflict Scale
IPPA-R: Inventory of Parent and Peer Attachment-Revised version
DSM–5: Diagnostic and Statistical Manual of Mental Disorders
SEM: Structural equation modeling
AVE: Average variance extracted
CR: Construct reliability
GFI: Goodness-of-fit index
RMSEA: Root mean square error of approximation
TLI: Tucker–Lewis Index
NFI: Normed fit index
CFI: Comparative fit index
AGFI: Adjusted goodness-of-fit index

## References

[1] Balogh KN, Mayes LC, Potenza MN. Risk-taking and decision-making in youth: relationships to addiction vulnerability. J Behav Addict. 2013;2(1). 10.1556/JBA.2.2013.1.1. 10.1556/JBA.2.2013.1.1 PMC3840427 24294500

[2] Davies EB, Morriss R, Glazebrook C (2014). Computer-delivered and web-based interventions to improve depression, anxiety, and psychological well-being of university students: a systematic review and meta-analysis. J Med Internet Res.

[3] Cummings EM, Schatz JN (2012). Family conflict, emotional security, and child development: translating research findings into a prevention program for community families. Clin Child Fam Psychol Rev.

[4] Keller PS, Gilbert LR, Koss KJ, Cummings EM, Davies PT (2011). Parental problem drinking, marital aggression, and child emotional insecurity: a longitudinal investigation. J Stud Alcohol Drugs.

[5] George MW, Fairchild AJ, Mark Cummings E, Davies PT (2014). Marital conflict in early childhood and adolescent disordered eating: emotional insecurity about the marital relationship as an explanatory mechanism. Eat Behav.

[6] Schneider LA, King DL, Delfabbro PH (2017). Family factors in adolescent problematic internet gaming: a systematic review. J Behav Addict.

[7] Wu CST, Wong HT, Yu KF, Fok KW, Yeung SM, Lam CH, Liu KM (2016). Parenting approaches, family functionality, and internet addiction among Hong Kong adolescents. BMC Pediatr.

[8] Yu L, Shek DT (2013). Internet addiction in Hong Kong adolescents: a three-year longitudinal study. J Pediatr Adolesc Gynecol.

[9] Kalaitzaki AE, Birtchnell J (2014). The impact of early parenting bonding on young adults' internet addiction, through the mediation effects of negative relating to others and sadness. Addict Behav.

[10] Tan ES, McIntosh JE, Kothe EJ, Opie JE, Olsson CA (2018). Couple relationship quality and offspring attachment security: a systematic review with meta-analysis. Attach Hum Dev.

[11] Bowlby J (1977). The making and breaking of affectional bonds. I. Aetiology and psychopathology in the light of attachment theory. An expanded version of the Fiftieth Maudsley Lecture, delivered before the Royal College of Psychiatrists, 19 November 1976. Br J Psychiatry.

[12] Bowlby J (1977). The making and breaking of affectional bonds. II. Some principles of psychotherapy. The fiftieth Maudsley lecture. Br J Psychiatry.

[13] Liu QX, Fang XY, Zhou ZK, Zhang JT, Deng LY (2013). Perceived parent-adolescent relationship, perceived parental online behaviors and pathological internet use among adolescents: gender-specific differences. PLoS One.

[14] Higgins GE, Jennings WG, Mahoney M (2010). Developmental trajectories of maternal and paternal attachment and delinquency in adolescence. Deviant Behav.

[15] Veselska Z, Geckova AM, Orosova O, Gajdosova B, van Dijk JP, Reijneveld SA (2009). Self-esteem and resilience: the connection with risky behavior among adolescents. Addict Behav.

[16] Ethier KA, Kershaw TS, Lewis JB, Milan S, Niccolai LM, Ickovics JR (2006). Selfesteem, emotional distress and sexual behavior among adolescent females: Interrelationships and temporal effects. J Adolesc Health.

[17] Jeynes W (2015). A meta-analysis: the relationship between father involvement and student academic achievement. Urban Educ.

[18] Zurita-Ortega F, Chacon-Cuberos R, Castro-Sanchez M, Gutierrez-Vela FL, Gonzalez-Valero G. Effect of an Intervention Program Based on Active Video Games and Motor Games on Health Indicators in University Students: A Pilot Study. Int J Environ Res Public Health. 2018;15(7). 10.3390/ijerph15071329. 10.3390/ijerph15071329 PMC6068999 29941811

[19] King DL, Delfabbro PH (2014). Is preoccupation an oversimplification? A call to examine cognitive factors underlying internet gaming disorder. Addiction (Abingdon, England).

[20] Lemmens JS, Valkenburg PM, Peter J (2011). The effects of pathological gaming on aggressive behavior. J Youth Adolesc.

[21] Jeong H, Yim HW, Jo SJ, Lee SY, Kim E, Son HJ, Han HH, Lee HK, Kweon YS, Bhang SY (2017). Study protocol of the internet user cohort for unbiased recognition of gaming disorder in early adolescence (iCURE), Korea, 2015-2019. BMJ Open.

[22] Grych JH, Seid M, Fincham FD (1992). Assessing marital conflict from the child's perspective: the children's perception of interparental conflict scale. Child Dev.

[23] Kwon YO, Lee JD (1997). A validation study on the Children's perception of Interparental conflict scale. Korean J Child Studies.

[24] Gao T, Meng X, Qin Z, Zhang H, Gao J, Kong Y, Hu Y, Mei S (2018). Association between parental marital conflict and internet addiction: a moderated mediation analysis. J Affect Disord.

[25] Armsden GC, Greenberg MT (1987). The inventory of parent and peer attachment: individual differences and their relationship to psychological well-being in adolescence. J Youth Adolesc.

[26] Ok J: Relationship between attachment security and depression in adolescence: focusing on the mediating effect of perceived competence. (Master’s thesis). Retrieved from http://www.riss.kr.access.yonsei.ac.kr 1998.

[27] Rosenberg M (1965). Society and the adolescent self-image.

[28] Jon BJ (1974). Self-esteem: a test of its measurability. Yonsei J.

[29] Jo SJ, Yim HW, Lee HK, Lee HC, Choi JS, Baek KY: The Internet Game Use-Elicited Symptom Screen proved to be a valid tool for adolescents aged 10–19 years. Acta paediatrica (Oslo, Norway : 1992) 2017. 10.1111/apa.14087 28940637

[30] Fornell CaL D (1981). Evaluating structural equation models with unobservable variables and measurement error. J Mark Res.

[31] Cronbach L (1951). Coefficinent alpha and the internal structure of tests. Psychometrika.

[32] Erceg-Hurn DM, Mirosevich VM (2008). Modern robust statistical methods: an easy way to maximize the accuracy and power of your research. Am Psychol.

[33] West SG, Taylor AB, Wu W, Hoyle RH (2012). Model fit and model selection in structural equation modeling. Handbook of structural equation modeling (pp. 209–231).

[34] De Leo JA, Wulfert E (2013). Problematic internet use and other risky behaviors in college students: an application of problem-behavior theory. Psychol Addict Behav.

[35] Yen JY, Yen CF, Chen CC, Chen SH, Ko CH (2007). Family factors of internet addiction and substance use experience in Taiwanese adolescents. Cyberpsychol Behav.

[36] Miller P, Plant M (2010). Parental guidance about drinking: relationship with teenage psychoactive substance use. J Adolesc.

[37] Shek DT (2002). Family functioning and psychological well-being, school adjustment, and problem behavior in chinese adolescents with and without economic disadvantage. J Genet Psychol.

[38] Ko CH, Yen JY, Chen CS, Yeh YC, Yen CF (2009). Predictive values of psychiatric symptoms for internet addiction in adolescents: a 2-year prospective study. Arch Pediatr Adolesc Med.

[39] Ablow JC, Measelle JR, Cowan PA, Cowan CP (2009). Linking marital conflict and children's adjustment: the role of young children's perceptions. J Family Psychol.

[40] Pinto A, Verissimo M, Gatinho A, Santos AJ, Vaughn BE (2015). Direct and indirect relations between parent-child attachments, peer acceptance, and self-esteem for preschool children. Attach Hum Dev.

[41] Whitney S, Prewett S, Wang Z, Chen H (2017). Fathers’ influence on Children’s cognitive and Behavioural functioning: a longitudinal study of Canadian families. Int J Child Youth Family Studies.

[42] Stafford M, Kuh DL, Gale CR, Mishra G, Richards M (2016). Parent-child relationships and offspring's positive mental wellbeing from adolescence to early older age. J Posit Psychol.

[43] Lee A: Does Self-Esteem Mediate the Effect of Attachment on Relationship Quality. All Theses and Dissertations 6420 https://scholarsarchive.byu.edu/etd/6420 2016.

[44] Su B, Yu C, Zhang W, Su Q, Zhu J, Jiang Y (2018). Father-child longitudinal relationship: parental monitoring and internet gaming disorder in Chinese adolescents. Front Psychol.

[45] Kim E, Yim HW, Jeong H, Jo SJ, Lee HK, Son HJ, Han HH (2018). The association between aggression and risk of internet gaming disorder in Korean adolescents: the mediation effect of father-adolescent communication style. Epidemiol Health.

[46] Deng LY, Zhang JT, Fang XY, Liu QX, Tang HY, Lan J (2012). Perceived parental conflict and adolescents' internet addiction: the mediating effect of adolescents' conflict appraisal and emotional management. Psychol Dev Educ.

[47] Eichenberg C, Schott M, Decker O, Sindelar B (2017). Attachment style and internet addiction: an online survey. J Med Internet Res.

[48] KISDI: Korean media panel survey. Korea Information Society Development Institute 2013, http://kisdi.re.kr/kisdi/jsp/fp/eng/main.jsp.

[49] Jeong H, Yim HW, Lee SY, Lee HK, Potenza MN, Kwon JH, Koo HJ, Kweon YS, Bhang SY, Choi JS (2018). Discordance between self-report and clinical diagnosis of internet gaming disorder in adolescents. Sci Rep.

